# Suppression of defense gene expression under nutrient-rich condition in *Arabidopsis* seedlings

**DOI:** 10.5511/plantbiotechnology.24.0726a

**Published:** 2024-12-25

**Authors:** Tetsutaro Nakamura, Yukiko Osawa, Rieko Ogura, Kazuyuki Hiratsuka

**Affiliations:** 1 Graduate School of Environment and Information Sciences, Yokohama National University, 79-7 Tokiwadai, Hodogayaku, Yokohama, Kanagawa 240-8501, Japan; 2 Yokohama Bio Technology Company Limited, 79-1 Tokiwadai, Hodogaya-ku, Yokohama, Kanagawa 240-8501, Japan

**Keywords:** *Arabidopsis thaliana*, firefly luciferase, high-throughput screening, plant activator

## Abstract

Plant hormones like salicylic acid (SA) and jasmonic acid (JA) play crucial roles in regulating defense gene expression systems. SA mainly regulates defense against biotrophic pathogens, while JA mediates defense against necrotrophic pathogens. Compounds called plant activators including probenazole, acibenzolar-s-methyl and 2,6-dichloroisonicotinic acid (INA) activate plant immune systems, providing protection against pathogens. Unlike conventional pesticides that directly target pathogens, plant activators boost the host’s defense mechanisms, potentially reducing the likelihood of drug resistance development. Various high-throughput screening systems (HTS) have been developed with the aim of searching for plant activators. Transgenic *Arabidopsis* lines expressing luciferase under the control of defense gene promoters allow us to monitor the activity of defense-related gene in vivo. To investigate the influence of nutrients on the HTS system, we conducted luciferase assays using *Arabidopsis* seedlings and observed the suppression of defense gene expression in response to the treatment of plant activators. Reverse transcription quantitative polymerase chain reaction (RT-qPCR) was employed to monitor the expression levels of endogenous genes in response to nutrient-rich conditions and confirmed the suppression effect of defense gene expression as observed in the luciferase reporter assays. The findings highlight the importance of considering nutrient effects when evaluating plant activators and screening for compounds that induce defense gene expression under nutrient-rich conditions.

Plants produce a variety of hormones, including auxin, gibberellins, abscisic acid, cytokinins, salicylic acid (SA), ethylene, jasmonic acid (JA), brassinosteroids and peptides, which are responsible for various abiotic and non-stress responses, as well as playing important roles in diverse growth and developmental processes (Bari and Jones 2009). Defense gene expression systems of higher plants responsible for biotic stresses are mainly regulated under the control of SA and JA-mediated pathways and control many downstream defense related genes. The class of compounds called plant activators, such as probenazole (PBZ), acibenzolar-s-methyl (ASM) and 2,6-dichloroisonicotinic acid (INA), protect plants from pathogens by activating the plant immune system (Görlach et al. 1996; Lawton et al. 1996; Yoshioka et al. 2001). Whereas commonly used pesticides directly target pathogens, plant activator is targeted to host defense system and has been shown to be less likely to develop drug resistance (Walters et al. 2013).

It has been shown that different environmental conditions such as light and nutrition influence defense gene expression and the efficacy of plant activators. Furthermore, it is known that the relationship between nitrogen supply and disease response gene expression in plants varies according to plant growth stage. Many of the previous studies have linked plant nutritional status to disease resistance, most of them have been conducted using mature plants. Therefore, the relationship between nutritional status and disease-responsive genes is not necessarily clear in the early stages of plant growth (Sun et al. 2020; Verly et al. 2020).

Various screening systems have been developed and evolved to efficiently search for plant activators or defense gene regulators (Narusaka et al. 2006; Noutoshi et al. 2012; Seo et al. 2012). Previously, we developed a high-throughput screening (HTS) system using bioluminescent reporter to select plant activators from various sources (Kusama et al. 2012; Minami et al. 2011; Ono et al. 2011; Watakabe et al. 2001). In this HTS system, a fusion gene linked to the firefly luciferase (firefly luciferase: Fluc) gene from *Photinus pyralis* was introduced to monitor the promoter activity of defense genes by bioluminescence detection.

The luciferase-reporter gene based HTS system with *Arabidopsis* seedlings initially used distilled water (DW) as the medium (Kusama et al. 2012; Ono et al. 2011). However, longer-term gene expression monitoring of the system and the application of transient gene expression by agroinfiltration require the use of nutrient-rich media (Wu et al. 2014). Therefore, in this study, to investigate the effect of nutrient addition on defense-responsive gene expression, we conducted a series of experiments using *Arabidopsis* seedlings.

First, we tested the inducibility of the tobacco *PR1a* (*NtPR1a*) promoter, a monitoring system for SA-dependent SAR-related responsiveness (Ono et al. 2004; Watakabe et al. 2011), in response to the treatment of the defense gene inducers SA (Sigma-Aldrich, St Louis, MO, USA), ASM (Wako, Osaka, Japan), PBZ (Wako), and INA (Aldrich). Transgenic *Arabidopsis* seeds harboring *PR-1a::Fluc* were sown aseptically on 96-well white plate (Grainer, Frickenhausen, Germany) with 89 µl of distilled water or nutrition rich liquid medium consisting of half concentration of Murashige and Skoog (MS) plant salt mixture (Wako) without sucrose (Murashige and Skoog 1962) and grown for 5 days at 22°C under continuous light. On day 5, 10 µl of 0.5 mM D-luciferin (Promega, Madison, WI, USA) was dispensed per well, and on the following day, the compounds SA, ASM, PBZ, INA and the solvent for these compounds, dimethylsulphoxide (DMSO) were dispensed in 1.0 µl per well to a final concentration of 30 µM for SA, ASM and INA, and 100 µM for PBZ, respectively. For monitoring of bioluminescence activity, images were captured using WSE-6300 Lumino Graph III (ATTO, Tokyo, Japan), and image analysis was performed using analysis software CS Analyzer 4 (ATTO).

Time-course measurement of bioluminescence levels from *Arabidopsis* seedlings showed that in DW, a clear induction of expression was observed after each treatment, with a minimum of 10-fold or more induction levels and a clear expression induction pattern was observed (Figure 1A). On the other hand, no induction of bioluminescence levels was observed in the nutrient-rich medium containing MS salts in each treatment and only a 2-fold or less induction was detected at later stage (168 h) of observation (Figure 1B). These results indicate that the presence of nutrient salts may cause strong repression of defense gene expression in at least some of the SA-responsive genes.

**Figure 1:**
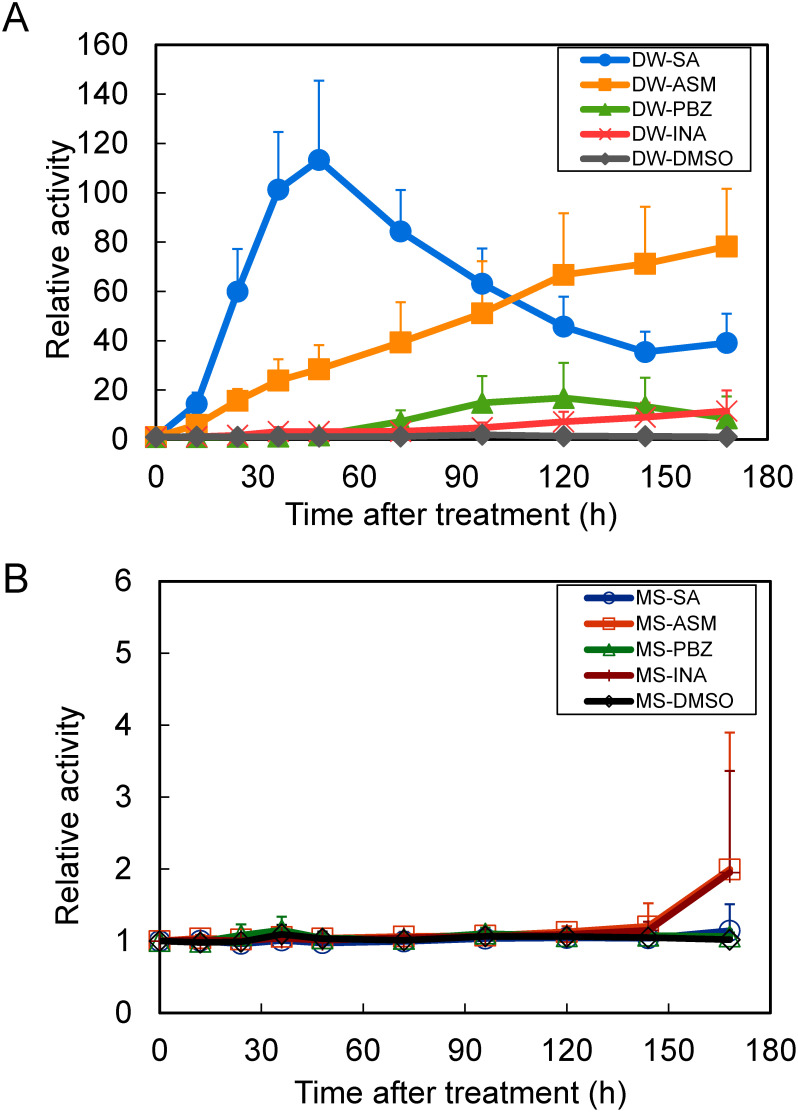
Figure 1. Monitoring of SA-responsive luciferase reporter activities in transgenic *Arabidopsis* seedlings under different nutritional condition. (A) Time-course measurement of bioluminescence levels from *PR-1a::Fluc* seedlings in distilled water (DW) after treatment with SA, ASM, PBZ, INA or DMSO (control). (B) Time-course measurement of bioluminescence levels from *PR-1a::Fluc* seedlings in 1/2MS liquid medium after treatment with SA, ASM, PBZ, INA or DMSO (control). Values are means±SD for six independent measurements.

To eliminate possible influence of nutrient salts on luciferase activity, we conducted the same experiment using *Arabidopsis* seedlings constitutively express Fluc under the control of the CaMV 35S promoter (*35S::Fluc*) (Matsuo et al. 2001). After the treatment, the Fluc expression was monitored by bioluminescence detection. As shown in Figure 2, up to two-fold induction of the Fluc activity was shown in response to treatments probably due to the induction effect of SA on the CaMV 35S promoter as described previously (Qin et al. 1994), no remarkable difference in expression levels was observed between DW and MS nutrient medium. These results suggest that the suppression of luciferase expression induction observed in Figure 1 is dependent on the *PR-1a* gene promoter activity.

**Figure 2:**
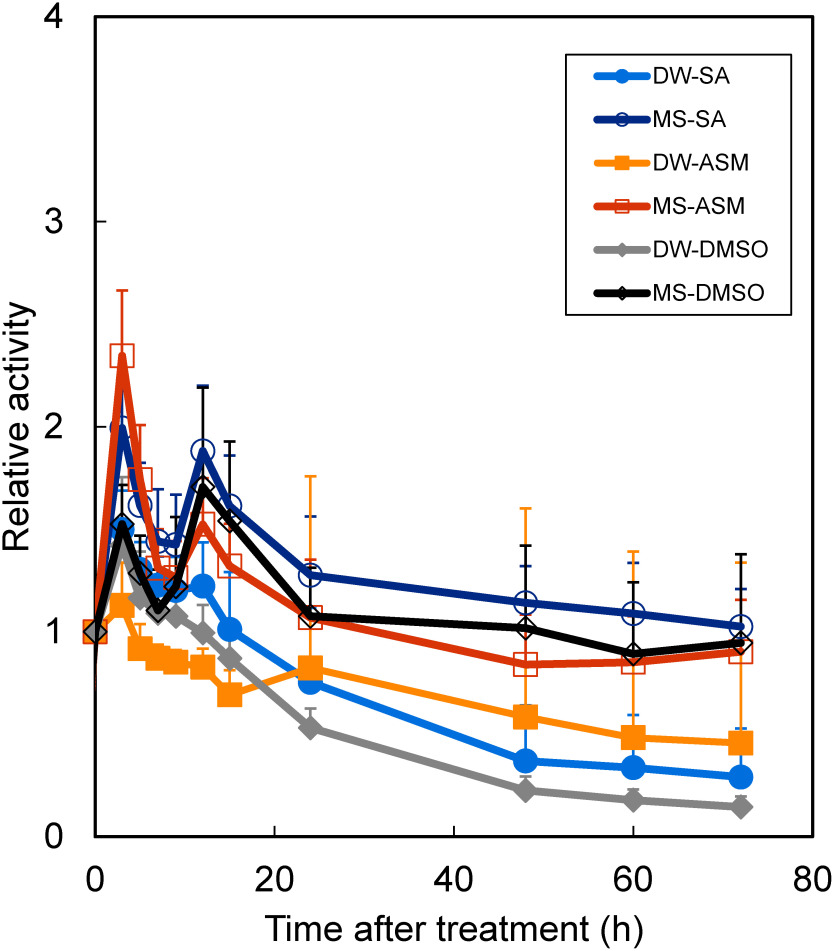
Figure 2. Monitoring of *35S::Fluc* activities in transgenic *Arabidopsis* seedlings under different nutritional condition. Time-course measurement of bioluminescence levels from *35S::Fluc* seedlings in DW or 1/2MS after treatment with SA, ASM or DMSO (control). Values are means±SD for six independent measurements.

Next, to investigate the non-SA defense gene expression we tested the influence of nutritional conditions on the JA mediated gene expression. We chose to use *Arabidopsis* seedlings harboring *Arabidopsis Vegetative Storage Protein 1* (*VSP1*) promoter-luciferase reporter gene as described previously (Kusama et al. 2012). Similar to the experimental procedure on the *NtPR-1a* promoter described above, transgenic *Arabidopsis* seedlings harboring *VSP1::Fluc* were grown in 96 well plates for one week and were treated with MeJA (Wako) or prohydrojasmonate (PDJ) (Wako), to a final concentration of 30 µM. After compound treatment, the measurement of luminescence activity was performed as described above. The results showed that, compared to MeJA, the JA derivative PDJ was observed to be less active in the MS culture. Compared to the plants cultured in DW, those cultured in MS medium showed an earlier timing of induction of expression and reduction of activity by MeJA and PDJ treatment. Although the higher activity was observed in the MeJA treatment when incubated in DW, this high activity was not maintained under nutrient-rich conditions (Figure 3).

**Figure 3:**
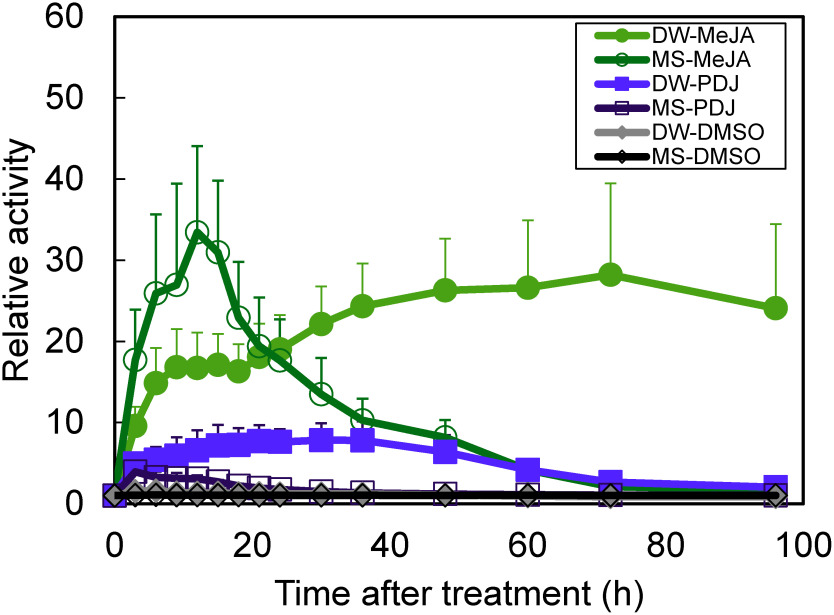
Figure 3. Monitoring of JA-responsive luciferase reporter activities in transgenic *Arabidopsis* seedlings under different nutritional condition. Time-course measurement of bioluminescence levels from *VSP1::Fluc* seedlings in DW or 1/2MS after treatment with MeJA, PDJ or DMSO (control). Values are means±SD for six independent measurements.

These results indicate that the defense response of the JA system is also altered under nutrient-rich conditions. In combination with the experiments focusing on the *NtPR-1a* promoter expression described above, it is possible that the expression of defense response-related genes is under the strong influence of nutritional conditions in *Arabidopsis* seedlings. These results suggest that screening for JA-responsive agents, as with SA-responsive agents, requires consideration of the effects of eutrophic conditions and media composition.

To investigating the expression levels of endogenous genes in response to nutrition-rich condition, the induction of expression of the *Arabidopsis* *PR-1* was monitored using RT-qPCR. Wild-type *Arabidopsis* (Col-0) seedlings grown for one week in 96 well plates were treated with SA or ASM at a final concentration of 30 µM. Total RNA was extracted using the RNeasy Plant mini kit (Qiagen, Hilden, Germany) and PrimeScript™ RT reagent kit with gDNA Eraser (TaKaRa, Shiga, Japan) was used for gDNA removal and cDNA synthesis by reverse transcription. The expression levels of the endogenous *Arabidopsis* *PR-1* were then quantified by RT-qPCR using TB Green® Premix Ex Taq™ II (TaKaRa) and Stratagene Mx3000P (Agilent Technologies, Santa Clara, CA, USA)

The qRT-PCR was performed using the primers PR1-FW (5′-CTAACTACAACTACGCTGCGAAC-3′), PR1-RV (5′-TTCATTAGTATGGGCTTCTCGTTCA-3′) and At1G13320-FW (5′-TAACGTGGCCAAAATGATGC-3′) and At1G13320-RV (5′-GTTCTCCACAACCGCTTGGT-3′). The RT-qPCR conditions were one cycle of temperature 95°C for 30 s for initial denaturation, followed by 40 amplification cycles of denaturation temperature 95°C for 5 s and elongation/annealing temperature 60°C for 30 s.

The results suggest that similar to the expression profiles obtained using the *NtPR-1a* promoter-reporter assay, the expression of the endogenous *PR-1* is suppressed in seedlings grown under MS liquid medium (Figure 4). Although the activation of the *NtPR-1a* promoter in response to treatment with chemicals was almost completely suppressed when plants are grown on MS liquid medium, some induction of expression of the *PR-1* mRNA was observed. This may be due to the fact that the *NtPR-1a* promoter from tobacco is more tightly regulated in its expression than the *Arabidopsis PR-1* promoter and is more susceptible to repressive factors in *Arabidopsis* seedlings (Ono et al. 2011). These results confirm that the suppression of disease-responsive gene expression in *Arabidopsis* seedlings identified in the present study is not due to the luciferase reporter assay, but is an actual phenomenon occurring in seedlings germinated and grown in nutrient-rich conditions.

**Figure 4:**
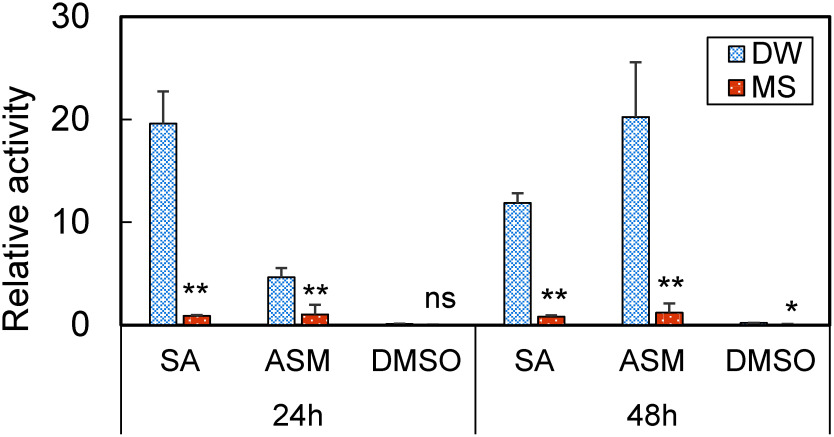
Figure 4. Comparison of responsiveness of *Arabidopsis* endogenous *PR-1* gene expression under different nutritional condition. Seven-day-old *Arabidopsis* seedlings grown in 96 well plates were treated with SA, ASM, and DMSO, respectively, as described in the text. Samples were collected after 24 and 48 h, and gene expression was quantified by RT-qPCR. The vertical axis represents relative expression level of the *PR-1* gene (*AT2G14610*) compared to the housekeeping gene (*At1G13320*). Values are means ±SD for three independent samples. Statistical significance in comparison with DW was determined by Student’s *t*-test (ns, not significant; * *p*<0.05; ** *p*<0.01).

To investigate the causes of these phenomena, a comparison of the metabolism, especially photosynthetic activity, of *Arabidopsis* seedlings grown on DW and MS media is needed. However, due to limited experimental conditions, these investigations could not be carried out in this study.

In previous studies, the relationship between nitrate fertilization and defense response gene expression has been investigated in considerably in detail (Verly et al. 2020). However, those findings were for mature leaves, and no data were presented for the germinated seedlings in multi-well plates used for HTS. In this study we show that in *Arabidopsis* seedlings, the nutritional status influences repression of at least one of the defense responsive gene expression mediated by SA or JA, which can be assumed to affect disease responsiveness and resistance. It can be concluded that this needs to be taken into account in the evaluation and screening of plant activators or defense gene modulators. On the other hand, this phenomenon can be used in the systematic search for compounds or factors that can effectively induce the activation of defense responsive genes even under eutrophic conditions. These findings can be applied to the HTS technology for plant activators with new desirable characteristics.

## Abbreviations

ASM: acibenzolar-s-methyl
CaMV: cauliflower mosaic virus
Fluc: firefly luciferase
HTS: high-throughput screening
JA: jasmonic acid
PR: pathogenesis related
*VSP1*: *vegetative storage protein 1*

## References

[RBari2009] Bari R, Jones JDG (2009) Role of plant hormones in plant defence responses. *Plant Mol Biol* 69: 473–488 19083153 10.1007/s11103-008-9435-0

[d67e479] Görlach J, Volrath S, Knauf-Beiter G, Hengy G, Beckhove U, Kogel KH, Oostendorp M, Staub T, Ward E, Kessmann H, et al. (1996) Benzothiadiazole, a novel class of inducers of systemic acquired resistance, activates gene expression and disease resistance in wheat. *Plant Cell* 8: 629–643 8624439 10.1105/tpc.8.4.629 PMC161125

[RKusama2012] Kusama M, Urata N, Ogura R, Ogata S, Hiratsuka K (2012) Development of a promoter-luciferase-based high-throughput system to monitor jasmonate-mediated defense gene expression. *Plant Biotechnol (Tokyo)* 29: 515–520

[RLawton1996] Lawton KA, Friedrich L, Hunt M, Weymann K, Dalaney T, Kessmann H, Staub T, Ryals J (1996) Benzothiadiazole induces disease resistance in *Arabidopsis* by activation of the systemic acquired resistance signal transduction pathway. *Plant J* 10: 71–82 8758979 10.1046/j.1365-313x.1996.10010071.x

[RMatsuo2001] Matsuo N, Minami M, Maeda T, Hiratsuka K (2001) Dual luciferase assay for monitoring gene expression in higher plants. *Plant Biotechnol (Tokyo)* 18: 71–75

[RMinami2011] Minami T, Tanaka T, Takasaki S, Kawamura K, Hiratsuka K (2011) In vivo bioluminescence monitoring of defense gene expression in response to treatment with yeast cell wall extract. *Plant Biotechnol (Tokyo)* 28: 481–484

[RMurashige1962] Murashige T, Skoog FK (1962) A revised medium for rapid growth and bio-assays with tobacco tissue cultures. *Physiol Plant* 15: 473–497

[RNarusaka2006] Narusaka M, Abe H, Kobayashi M, Kubo Y, Kawai K, Izawa N, Narusaka Y (2006) A model system to screen for candidate plant activators using an immune-induction system in *Arabidopsis.* *Plant Biotechnol (Tokyo)* 23: 321–327

[RNoutoshi2012] Noutoshi Y, Okazaki M, Kida T, Nishina Y, Morishita Y, Ogawa T, Suzuki H, Shibata D, Jikumaru Y, Hanada A, et al. (2012) Novel plant immune-priming compounds identified via high-throughput chemical screening target salicylic acid glucosyltransferases in *Arabidopsis.* *Plant Cell* 24: 3795–3804 22960909 10.1105/tpc.112.098343 PMC3480303

[ROno2011] Ono S, Kusama M, Ogura R, Hiratsuka K (2011) Evaluation of the use of the tobacco *PR-1a* promoter to monitor defense gene expression by the luciferase bioluminescence reporter system. *Biosci Biotechnol Biochem* 75: 1796–1800 21897029 10.1271/bbb.110326

[ROno2004] Ono S, Tanaka T, Watakabe Y, Hiratsuka K (2004) Transient assay system for the analysis of *PR-1a* gene promoter in tobacco BY-2 cells. *Biosci Biotechnol Biochem* 68: 803–807 15118306 10.1271/bbb.68.803

[RQin1994] Qin XF, Holuigue L, Horvath DM, Chua NH (1994) Immediate early transcription activation by salicylic acid via the cauliflower mosaic virus *as-1* element. *Plant Cell* 6: 863–874 8061520 10.1105/tpc.6.6.863 PMC160484

[RSeo2012] Seo EK, Nakamura H, Mori M, Asami T (2012) Screening and characterization of a chemical regulator for plant disease resistance. *Bioorg Med Chem Lett* 22: 1761–1765 22260769 10.1016/j.bmcl.2011.12.082

[RSun2020] Sun Y, Wang M, Mur LAJ, Shen Q, Guo S (2020) Unravelling the roles of nitrogen nutrition in plant disease defences. *Int J Mol Sci* 21: 572 31963138 10.3390/ijms21020572 PMC7014335

[RVerly2020] Verly C, Djoman ACR, Rigault M, Giraud F, Rajjou L, Saint-Macary M-E, Dellagi A (2020) Plant defense stimulator mediated defense activation is affected by nitrate fertilization and developmental stage in *Arabidopsis thaliana.* *Front Plant Sci* 11: 583 32528493 10.3389/fpls.2020.00583 PMC7264385

[RWalters2013] Walters DR, Ratsep J, Havis ND (2013) Controlling crop diseases using induced resistance: Challenges for the future. *J Exp Bot* 64: 1263–1280 23386685 10.1093/jxb/ert026

[RWatakabe2001] Watakabe Y, Ono S, Hiratsuka K (2001) Characterization of agents that induce acquired resistance by transgenic plants. *J Pestic Sci (Nihon Nouyaku Gakkaishi)* 26: 296–299 (in Japanese)

[RWatakabe2011] Watakabe Y, Ono S, Tanaka T, Hiratsuka K (2011) Non-destructive bioluminescence detection system for monitoring defense gene expression in tobacco BY-2 cells. *Plant Biotechnol (Tokyo)* 28: 295–301

[RWu2014] Wu HY, Liu KH, Wang YC, Wu JF, Chiu WL, Chen CY, Wu SH, Sheen J, Lai EM (2014) AGROBEST: An efficient *Agrobacterium*-mediated transient expression method for versatile gene function analyses in *Arabidopsis* seedlings. *Plant Methods* 10: 19 24987449 10.1186/1746-4811-10-19 PMC4076510

[RYoshioka2001] Yoshioka K, Nakashita H, Klessig DF, Yamaguchi I (2001) Probenazole induces systemic acquired resistance in *Arabidopsis* with a novel type of action. *Plant J* 25: 149–157 11169191 10.1046/j.1365-313x.2001.00952.x

